# Small Intestine Ultrasound Findings on Horses Following Exploratory Laparotomy, Can We Predict Postoperative Reflux?

**DOI:** 10.3390/ani9121106

**Published:** 2019-12-09

**Authors:** Gabriel Cuevas-Ramos, Lara Domenech, Marta Prades

**Affiliations:** 1 Large Animal Clinic, Copenhagen University, Agrovej 8, 2630 Taastrup, Denmark; 2 Campus UAB, Universitat Autònoma de Barcelona, 08193 Bellaterra, Spain; lara.domenechd@e-campus.uab.cat (L.D.); marta.prades@uab.cat (M.P.)

**Keywords:** exploratory laparotomy, abdominal ultrasound, horses, postoperative reflux

## Abstract

**Simple Summary:**

Postoperative reflux is a well-recognized complication after exploratory laparotomy, particularly in horses that present with small intestine pathology. Even though much has been written about the pathophysiology and management of this postsurgical complication, we could not find a study that describes the monitoring of small intestine appearance after laparotomy via transcutaneous abdominal ultrasound. Therefore, the aim of the study was to provide clinical evidence of ultrasound finds in 58 horses over three days post exploratory laparotomy. The results from these exams were compared to the abdominal ultrasounds of 20 horses undergoing general anesthesia for an elective procedure, which were used as a control group. Differences were found between horses with versus without small intestinal pathology. Horses operated on because of large colon pathology had similar ultrasound findings to the control group during the postoperative period. In contrast, horses that were presented with small intestinal pathology had more visible small intestine loops, increased loop diameter, and wall thickness, before and after surgery, particularly those cases that had undergone a resection and anastomosis. A quick abdominal ultrasound in horses, during the postoperative period after colic surgery, was a useful method to identify horses with abnormal small intestinal parameters, both pre- and post-surgery. Further investigation as to whether these parameters can be used to predict postoperative reflux (POR) in a larger population is warranted.

**Abstract:**

Postoperative reflux (POR) is a well-recognized complication after colic surgery in horses, particularly when presenting small intestinal pathology. Even though much has been written about the pathophysiology and management of POR, additional clinical studies are needed to better understand and anticipate this complication. The aim of the study was to provide clinical evidence of ultrasound findings in the postoperative period (three days). The study is based on transcutaneous abdominal ultrasounds of the caudoventral abdomen during the postoperative period (three days), in 58 horses, presented for an exploratory laparotomy, and compared to 20 horses that underwent general anesthesia for an elective surgical procedure. Small intestine (SI) images and videos were analyzed for loop number, loop diameter, wall thickness, motility, and echogenic type of loop contents. Ultrasound findings of horses that had a large colon pathology were similar to those of the control group. Interestingly, horses that presented an SI pathology had significantly thicker SI walls, increased loop diameter, slower motility, and hypoechoic contents, particularly in horses that had undergone small intestinal resection and anastomosis. Although the number of horses that developed POR in our study was too small for statistical analysis, they all had the aforementioned ultrasonographic changes. Abdominal ultrasound, during the postoperative period (three days), was a useful method to identify horses with abnormal small intestinal parameters. Further investigation as to whether these parameters can be used to predict POR in a larger population is warranted.

## 1. Introduction

Postoperative reflux (POR) is a recognized complication that can lead to death or euthanasia during the postoperative period of horses undergoing an exploratory laparotomy, particularly those with small intestinal lesions. POR should not be confused with postoperative ileus (POI), as the latter is defined as a functional disturbance, with interruption of, or reduction in, gastrointestinal transit [1]. POI has the potential to significantly increase hospitalization time, treatment costs and postoperative morbidity, and mortality [2,3], and not all horses with POI will have reflux [4]; therefore, POR is probably multifactorial [1]. Commonly used criteria to identify horses with POR are intended to define cases in retrospective studies based almost exclusively on the volume of reflux [4,5]. Consequently, POR has been differently defined according to the amount of nasogastric reflux obtained within a certain period or a specific moment; high-volume (≥20 L) POR over 24 h, ≥8 L during a single nasogastric intubation, or >2 L of reflux at any selected time [6,7,8,9].

Transcutaneous abdominal ultrasonography has become an integral part of the diagnostic workup during an equine colic evaluation. An accurate assessment of horses with acute abdominal pain is essential for the establishment of a correct diagnosis, prognosis, and particularly for the decision to pursue either a medical or surgical management [10]. There is extensive information of common abdominal ultrasound findings on horses with an acute colic episode [11,12]. As a preoperative diagnostic tool, transcutaneous abdominal ultrasound has been reported to have 80% sensitivity and 96.15% specificity for small intestinal (SI) obstruction [13], and more importantly, when distended and non motile SI was detected, the sensitivity, specificity, and positive or negative predictive values for SI strangulation obstructions were of 100% [10,11]. The average normal SI wall thickness has been reported to be around <3 mm, with an average loop diameter of 2 cm [10,11], with reported abnormal measurements going up to 1.3 cm on wall thickness and 13.5 cm in loop diameter. These findings indicate that ultrasound is a useful diagnostic tool to aid in determining whether a horse needs colic surgery or not. A quick-and-easy protocol has also been reported [13]. The protocol divides the horse’s abdomen into seven regions that are assessed using alcohol saturation without clipping. This technique permitted a thorough abdominal examination in approximately 10 min and was able to show free peritoneal fluid and intestinal loop abnormalities.

When reviewing the current literature, even if there is extensive data on the use of transcutaneous abdominal ultrasound as a preoperative diagnostic tool for detecting SI pathologies [14], we could not find publications that report its use during the immediate postoperative period. Nevertheless, Sheats et al. [15] reported that large colon wall involution, evaluated during the immediate post-surgery period, was associated with decreased postoperative morbidity in horses presented for surgical correction of large colon volvulus without resection. While our long term hypothesis is that postsurgical transcutaneous abdominal ultrasound of the caudo-ventral abdomen could help identify horses at risk of developing POR, our short term objectives for this study were, to develop a standardized approach to ultrasound the SI in the horse post-laparotomy, and to obtain proof of principle data supporting differences in SI appearance and motility following colic surgery in horses with different types of intestinal lesions, as there is a lack of clinical data on this aspect.

## 2. Materials and Methods

**Figure 1:**
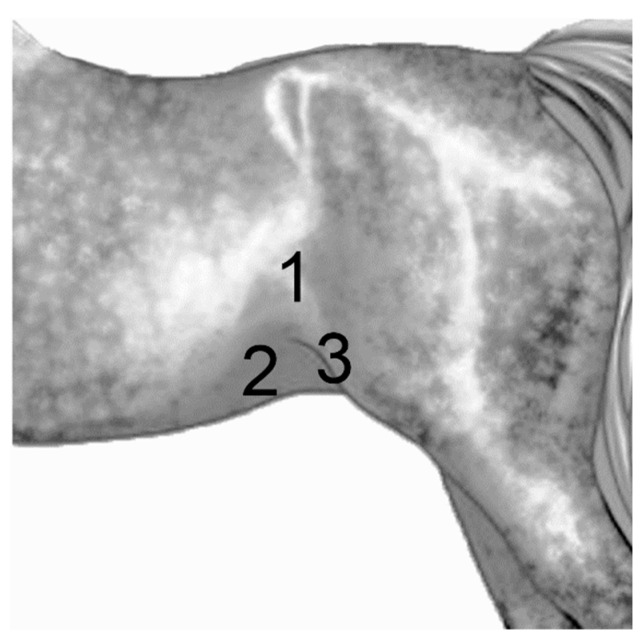
Ultrasound regions. Using a transcutaneous abdominal ultrasound protocol, three zones were systematically scanned in the caudo-ventral abdomen, of all cases included in the study. These regions allowed us to perform these exams even if the horse had an abdominal bandage protecting the surgical site. Left and right sides were examined. Flank (**1**), ventral (**2**), where the ultrasound probe was placed next to the midline and moved laterally, and inguinal (**3**), where the probe was placed medially to the stifle, in the most caudal part of the abdomen.

During a two year period, horses referred to the Hospital Clinic Veterinari (Equine Service) of the Universitat Autònoma de Barcelona (Barcelona, Spain), and that underwent an exploratory laparotomy, were included in the study if they had an abdominal ultrasound evaluation performed upon arrival to the hospital (before surgery), and also a daily abdominal ultrasound exam, for three consecutive days, postoperatively. Clinical cases lacking this requisite were excluded from the study. A total of 58 cases fulfilled these criteria. As a control group, 20 horses undergoing anesthesia for an elective surgical procedure (i.e., arthroscopy) were included, following the same ultrasound exam protocol, one before surgery and one per day the following three days after surgery. Abdominal ultrasounds were done by the same operator, with a convex transducer (3.5 MHz frequency), using the My Lab 70 Vision Esaote machine. Three abdominal areas were analyzed systematically in the caudo-ventral abdomen, referred to as ventral, inguinal, and flank regions (Figure 1), left and right sides. Image acquisition was as optimized as possible, trying to visualize as many SI loops as possible. Three images and one video were stored per region. Data were then pooled together per group and time of analysis. Observed small intestinal loops were recorded and analyzed for number, diameter (measured in centimeters), wall thickness (measured in millimeters), motility (normal peristalsis was considered as 6 to 15 contractions per minute [16]), and type of contents (either hyper or hypoechogenic). All postoperative ultrasound exams were performed at the same time in the morning (10:00), and the amount of time used for each exam was recorded. As the study did not mean to interfere with ongoing treatment decisions, the related therapeutic data were not considered as part of the study. Only the type of pathology during surgery and the presence or not of POR were included. For data analysis, exploratory laparotomy cases were divided into three groups according to the type of abdominal pathology found during surgery. Groups included the large colon (LC), small intestine with no resection (SInr), small intestine with resection and anastomosis (SIra), and the control group (Ct). Statistical analysis was performed with the GraphPad Prism 8 software, the statistical test ANOVA, Kruskal-Wallis, and Wilcoxon Rank Sum Test.

## 3. Results

In total, 78 horses were included in the study, 20 in the Ct group, 38 horses in the LC group, 12 in the SInr group, and 8 in the SIra group. The average age of the horses was 10 years; the younger one was 1.5 years old, and the eldest was 27 years old. There were 20 females, 25 geldings, and 13 males. A total of 1044 images and 348 videos were analyzed for each ultrasound time set: preoperative, 24, 48, and 72 h post-surgery. The average exam needed 12 min to be performed independently of the group.

**Figure 2:**
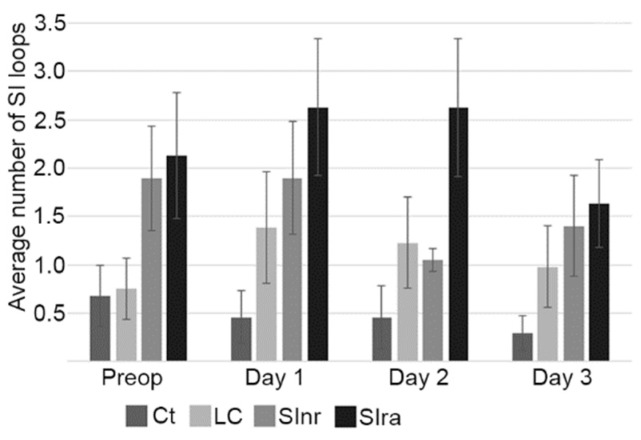
Number of small intestine loops. The Ct and LC groups had very similar findings on the first ultrasound exam (before surgery), post-surgery the LC group had higher counted SI loops than the Ct group, probably the result of hand manipulation. The groups where the small intestine was affected, SInr and SIra, presented higher numbers of visible SI loops, and this tendency remained along the postoperative period. Control (Ct), large colon (LC), small intestine no resection (SInr), and small intestine with resection and anastomosis (SIra). Preoperative ultrasound (Preop). Ultrasounds following three consecutive postoperative days (Day 1 to Day 3). Bars represent standard error of the mean.

When analyzing the number of SI loops counted per exam between groups, the Ct and LC groups had very similar findings on the first ultrasound exam (before surgery) (Figure 2). In the postoperative period, the Ct group remained with lower counted SI loops than the other groups. Interestingly, when analyzing only the LC group, the variation on the number of counted SI loops between the first ultrasound (pre-surgery) and the postoperative exams was of significant value (*p* = 0.0053), probably the result of hand manipulation. When analyzing all groups and exams, this parameter presented a high standard deviation, which could mean that the number of SI loops that can be observed and counted will vary a lot between cases, pre and post-surgery.

**Figure 3:**
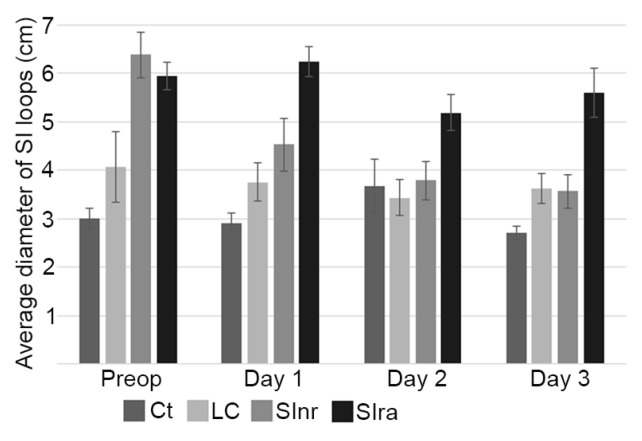
Diameter of small intestine loops. When comparing Ct and LC groups with the SInr and SIra groups, significant differences were observed, particularly on the first ultrasound exam (before surgery), and while the SInr group had smaller loop diameters during the postoperative period, the SIra group remained with elevated values. Control (Ct), large colon (LC), small intestine no resection (SInr), and small intestine with resection and anastomosis (SIra). Preoperative ultrasound (Preop). Ultrasounds following three consecutive postoperative days (Day 1 to Day 3). Bars represent standard error of the mean.

When analyzing the diameter of SI loops, there were no statistically significant differences between the Ct and LC groups. When comparing these two groups with the SInr and SIra groups, significant differences were observed, particularly on the first ultrasound exam (before surgery). Interestingly, the biggest diameters were observed in the SIra group, particularly during the three postoperative days (Figure 3). In this group, three cases had, on average, more than 6 cm in diameter, and all three presented some level of POR; two had >2 L per nasogastric check, and one had >8 L (nasogastric checks were done whenever the horse presented signs of increased pain). When analyzing differences within each group, the only one that had significant changes was the SInr group (*p* = 0.00017), as this group presented an important reduction of total diameter before and after surgery.

**Figure 4:**
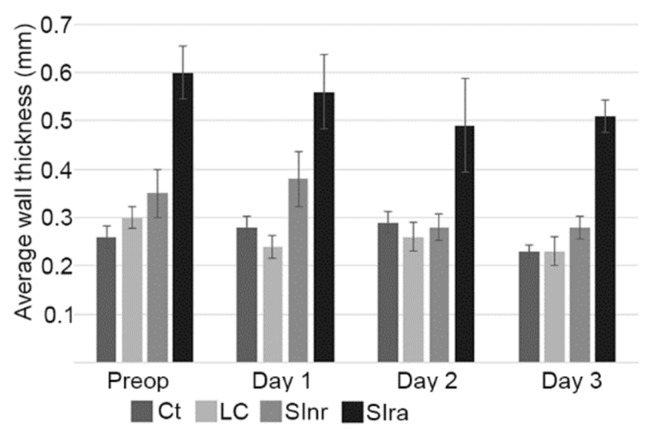
Small intestine wall thickness. Cases that presented higher wall thickness values, before and after surgery, were those that needed a resection and anastomosis (SIra group). The average wall thickness diminished during the postoperative period in the SInr group, but it remained significantly thicker (0.51 ± 0.04 cm) in the SIra group. This same group presented bigger diameter values. Control (Ct), large colon (LC), small intestine no resection (SInr), and small intestine with resection and anastomosis (SIra). Preoperative ultrasound (Preop). Ultrasounds following three consecutive postoperative days (Day 1 to Day 3). Bars represent standard error of the mean.

Wall thickness measurements presented a similar distribution than loop diameter. Similar values were observed between the Ct and LC groups, with statistical differences when compared to the SInr and SIra groups, particularly on day one after surgery. The average wall thickness diminished during the postoperative period in the SInr group, but it remained increased in the SIra group; moreover, wall thickness was significantly thicker (0.51 ± 0.04 cm) in the cases that also presented bigger loop diameter values (>5.59 ± 0.68 cm) and POR (Figure 3 and Figure 4).

SI motility and type of contents were analyzed with the videos taken during the ultrasound exams. Slower loop movement (<6 contractions per minute [16]) was observed only in the SIra group, together with hypoechogenic intestinal contents, including the three cases that presented POR. There was no significant difference between the Ct, LC, and SInr groups. 

## 4. Discussion

Measuring gastrointestinal parameters using a transcutaneous abdominal ultrasound approach, in the horse, has been proven to be a repeatable and reliable method [17], even when considering operator variabilities. This is important when interpreting abdominal ultrasound images, particularly when animals are reexamined by different subjects. In this study, flank, inguinal, and ventral regions (caudoventral abdomen) (Figure 1) were highly reliable in identifying SI loops, which is consistent with other publications [17,18]. Additionally, the selected areas were accessible for the ultrasound exam even if the horse had an abdominal bandage protecting the surgical site, which allowed us to pursue the study without interfering with the routine clinical management of the patients. The advantage of this is that our approach can be easily applied in equine hospitals where abdominal bandages are used during the postoperative period after an exploratory laparotomy.

It has been reported that general anesthesia reduces SI peristalsis for around 24 h [19]. The advantage of including a control group that underwent general anesthesia gave us the opportunity to include the possible effects of this episode and compare, via ultrasound examination, the effects of small intestinal manipulation. As the study did not mean to interfere with ongoing treatment decisions, the related therapeutic data were not considered as part of the analysis, which might be a limitation, but as the aim of the study was to obtain proof of principle data supporting differences in SI in the postoperative period, only the type of pathology during surgery and the presence or not of POR were included. Nonetheless, all ultrasounds done after surgery were scheduled at the same time (10:00) in order to homogenize the majority of cases to about the same postprandial moment, given that the first meal of the day was offered at around 07:00. As expected, some horses had reduced or no food intake post-surgery, particularly in the SIra group, but by doing the ultrasound examinations at the same time of the day we homogenized the majority of the cases in this matter without interfering with clinical treatments. Consequently, we could expect horses to have a similar amount and type of SI contents and similar stimuli for SI movement. As mention before, the total sample size included 1044 images and 348 videos per each time set (preoperative, 24, 48, and 72 h postoperative). After analyzing this, we observed minimal differences in terms of peristalsis, as slower loop movement was observed only in the SIra group. This was probably due to the lower number of cases in this group. Importantly, all the SIra cases (eight cases) had hypoechogenic SI contents, in contrast with the other groups where contents vary in echogenicity, but none were hypoechogenic. We could not find in the literature any description of what reflux should look like during an ultrasound examination, and with the low number of cases in the SIra group, it is hard to make any conclusions, but this should be investigated in a larger group of equine patients.

There were minimal differences between the Ct and LC groups. The most important differences were seen in the SInr and SIra groups when compared with the LC and Ct groups (Figure 2). During the studied postoperative period (three days), the higher values in wall thickness and loop diameter (Figure 3 and Figure 4) in the SIra group could indicate the ongoing inflammatory process of the SI, even after surgery. These findings have been documented before in the pre-surgical setting, as diagnostic signs of SI pathology [13,14], but there are no reports in the literature in terms of ultrasound findings and follow up during the immediate postoperative period. One study evaluated SI post-surgery when studying the effect of intravenous lidocaine, but only cases with SI pathology were included, and they were evaluated only 12 and 24 h post-surgery [20]. Our observations on the SIra (8 cases) also indicate that horses with the larger loop diameter, and thicker walls, also developed POR (3 cases). Unfortunately, the number of cases that could be included in the present study is low, and conclusions cannot be made. Nevertheless, this trend needs to be investigated, as these ultrasound findings could help identify horses at higher risk of developing POR.

Notably, we can conclude that horses that have undergone an exploratory laparotomy due to large colon pathology should be expected to have normal SI ultrasound findings during the postoperative period, and consequently, if distended SI loops or increased wall thickness is observed in LC cases, it could be indicative of another ongoing pathology.

## 5. Conclusions

This is the first study monitoring SI post abdominal surgery, for three consecutive days, with a transcutaneous ultrasound of the caudo-ventral abdomen. Horses operated on due to large colon pathology should be expected to have normal SI ultrasound parameters. Higher values of SI wall thickness and loop diameter are expected in horses that have suffered SI pathology; if there was no resection and anastomosis, SI values should get back to normal averages during the postoperative period (three days). Horses that underwent SI resection are expected to present abnormal ultrasound parameters during this period. A larger case compilation could help establish if postsurgical transcutaneous abdominal ultrasounds of the caudo-ventral abdomen could help identify horses at risk of developing POR.
